# Identification of Potential Vaccine Candidates Against SARS-CoV-2 to Fight COVID-19: Reverse Vaccinology Approach

**DOI:** 10.2196/32401

**Published:** 2022-04-26

**Authors:** Ekta Gupta, Rupesh Kumar Mishra, Ravi Ranjan Kumar Niraj

**Affiliations:** 1 Dr. B. Lal Institute of Biotechnology Jaipur India; 2 Amity University Rajasthan Jaipur India

**Keywords:** COVID-19, SARS-CoV-2, reverse vaccinology, molecular docking, molecular dynamics simulation, vaccine candidates, vaccine, simulation, virus, peptide, antigen, immunology, biochemistry, genetics

## Abstract

**Background:**

The recent emergence of COVID-19 has caused an immense global public health crisis. The etiological agent of COVID-19 is the novel coronavirus SARS-CoV-2. More research in the field of developing effective vaccines against this emergent viral disease is indeed a need of the hour.

**Objective:**

The aim of this study was to identify effective vaccine candidates that can offer a new milestone in the battle against COVID-19.

**Methods:**

We used a reverse vaccinology approach to explore the SARS-CoV-2 genome among strains prominent in India. Epitopes were predicted and then molecular docking and simulation were used to verify the molecular interaction of the candidate antigenic peptide with corresponding amino acid residues of the host protein.

**Results:**

A promising antigenic peptide, GVYFASTEK, from the surface glycoprotein of SARS-CoV-2 (protein accession number QIA98583.1) was predicted to interact with the human major histocompatibility complex (MHC) class I human leukocyte antigen (HLA)-A*11-01 allele, showing up to 90% conservancy and a high antigenicity value. After vigorous analysis, this peptide was predicted to be a suitable epitope capable of inducing a strong cell-mediated immune response against SARS-CoV-2.

**Conclusions:**

These results could facilitate selecting SARS-CoV-2 epitopes for vaccine production pipelines in the immediate future. This novel research will certainly pave the way for a fast, reliable, and effective platform to provide a timely countermeasure against this dangerous virus responsible for the COVID-19 pandemic.

## Introduction

COVID-19 began in December 2019 with an outbreak of a novel virus in Wuhan city of China [1]. The disease gained a rapid foothold worldwide, resulting in the World Health Organization (WHO) declaring it a global pandemic by March 2020. As of March 10, 2021, there has been a worldwide total of 118,159,602 cases and 2,622,101 deaths due to COVID-19 reported by the WHO. The virus causing COVID-19, SARS-CoV-2, spreads primarily through saliva, droplets, or discharges from the nose of an infected person after coughing or sneezing. Coronaviruses are enveloped RNA viruses with the largest genome among all RNA viruses [2]. As continuous transmission of the virus across borders increases, imposing a major health burden on the global scale, more studies are urgently required to understand SARS-CoV-2. Moreover, in the absence of effective cures and drugs, vaccination or immunization therapy is imperative to target the entire population. In particular, immunoinformatics tools have proven to be crucial to move the vaccine development pipeline forward [3]. Since there is relatively little knowledge about the pathogenesis of the virus, an immunoinformatics-based approach to investigate the immunogenic epitopes for further vaccine development is required [4].

Since COVID-19 has affected almost the entire world’s population, binding of promiscuous epitopes to a variety of human leukocyte antigen (HLA) alleles is vital for larger dissemination. Toward this end, in silico approaches will be remarkably useful in helping to develop a cure as quickly as possible [5]. Antibody generation by the activation of B cells as well as acute viral clearance by T cells along with virus-specific memory generation by CD8+ T cells are analogously important to develop immunity against the virus [6]. The SARS-CoV-2 spike (S) protein is considered to be highly antigenic, and thereby can evoke strong immune responses and generate neutralizing antibodies that can block attachment of the virus to host cells [7].

In reverse vaccinology, various in silico biology tools are used to discover novel antigens by studying the genetic makeup of a pathogen and the genes that could lead to identification of good epitopes. The reverse vaccinology approach thus offers a fast and cost-effective vaccine discovery platform [8]. With this approach, a novel antigen is identified using omics analysis of the target organism. In silico analysis combined with the reverse vaccinology approach facilitates an easier and time- and labor-saving process of antigen discovery [9].

**Figure 1:**
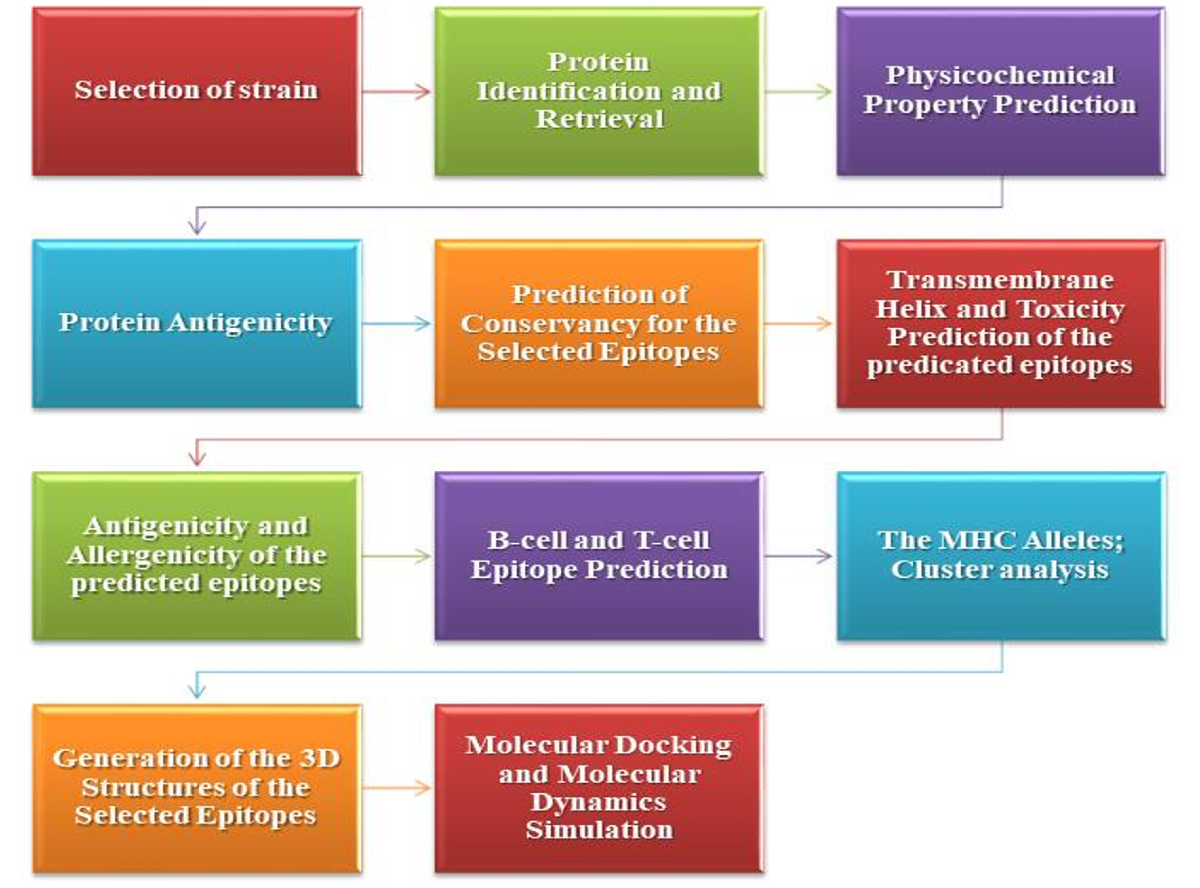
Diagrammatic representation of the methodology. MHC: major histocompatibility complex.

Herein, we explored the proteome of SARS-CoV-2 strains prominent in the Indian geographical region against the human host to identify potential antigenic proteins and epitopes that can effectively elicit a cellular-mediated immune response against the virus. With this approach, we identified a promising antigenic peptide, GVYFASTEK, from a surface glycoprotein (protein accession number QIA98583.1) of SARS-CoV-2, which was predicted to interact with human major histocompatibility complex (MHC) alleles and displayed up to 90% conservancy and significant antigenicity. Molecular docking analysis further confirmed the molecular interaction of the prime antigenic peptide with the residues of the HLA-A*11-01 allele for MHC class I. An overview of the study design is provided in Figure 1. After careful evaluation, this peptide was determined to be an appropriate epitope for eliciting a strong cell-mediated immune response against SARS-CoV-2. The outcomes from this significant analysis could help to select appropriate SARS-CoV-2 epitopes for multiepitope vaccine production pipelines in the near future. This novel research will certainly pave the way for a fast, reliable, and effective platform to provide a timely countermeasure against this dangerous pandemic disease.

## Methods

### Strain Selection

The highly virulent strain of SARS-CoV-2 was selected for the in silico analysis. The complete genome of SARS-CoV-2 is available in the National Center for Biotechnology Information database under reference NC_045512.2.

### Protein Identification and Retrieval

The following 12 viral protein sequences of SARS-CoV-2 were retrieved from the ViPR database (Host: Human, Country: India) [10]: Orf10 protein (QIA98591.1), Orf8 protein (QIA98589.1), Orf7a protein (QIA98588.1), Orf6 protein (QIA98587.1), Orf3a protein (QIA98584.1), membrane glycoprotein (QIA98586.1), envelope protein (QIA98585.1), surface glycoprotein (QIA98583.1), surface glycoprotein (QHS34546.1), nucleocapsid protein (QII87776.1), nucleocapsid protein (QII87775.1), and nucleocapsid phosphoprotein (QIA98590.1).

### Physicochemical Property Prediction

The online tool ProtParam of ExPASy [11] was used to predict various physicochemical properties of the selected protein sequences.

### Protein Antigenicity

VaxiJen v2.0 [12] was used to predict the antigenicity of the selected proteins. This software uses the FASTA file format of amino acid sequences as input and then predicts antigenicity based on the physicochemical properties of proteins. The output is denoted according to an antigenic score [13]. During analysis, the threshold was maintained at 0.4 [9].

### B Cell and T Cell Epitope Prediction

The B cell and T cell epitopes of the selected surface glycoprotein sequence were predicted via the Immune Epitope Database (IEDB), which contains a large amount of experimental data on epitopes and antibodies [14]. The IEDB enables performing a robust analysis on several epitopes in the context of various tools, including conservation across antigens, population coverage, and clusters with similar sequences [15]. To obtain MHC class I–restricted CD8+ cytotoxic T lymphocyte epitopes of the selected surface glycoprotein sequence, the NetMHCpan EL 4.0 prediction method was applied for the HLA-A*11-01 allele. MHC class II–restricted CD4+ helper T lymphocyte epitopes were obtained for the HLA DRB1*04-01 allele using the Sturniolo prediction method. The top 10 MHC class I and top 10 MHC class II epitopes were randomly selected based on their percentile scores and antigenicity scores. Five random B cell lymphocyte epitopes were selected based on their greater lengths using the Bipipered linear epitope prediction method [8].

### Antigenicity and Allergenicity of the Predicted Epitopes

VaxiJen v2.0 was utilized to predict protein antigenicity. During antigenicity analysis, the threshold was maintained at 0.4 [9]. The allergenicity of the selected epitopes was predicted via AllerTOP v2 [16].

### Transmembrane Helix and Toxicity Prediction of the Predicted Epitopes

The transmembrane helix of the selected epitopes was predicted using the TMHMM v2.0 server [17], which predicts whether the epitope would be in the transmembrane region, or remain inside or outside of the membrane. The toxicity prediction of the selected epitopes was carried out via the ToxinPred server [18].

### Prediction of Conservation of the Selected Epitopes

The conservation analysis of the epitopes was performed via the epitope conservancy analysis tool of the IEDB server [15]. During analysis, the sequence identity threshold was maintained at ≥50 [8].

### Cluster Analysis of MHC Alleles

Cluster analysis was carried out by MHCcluster 2.0 [19,20]. During cluster analysis, the number of peptides to be included was kept at 50,000 and the number of bootstrap calculations was set to 100. For cluster analysis, the NetMHCpan-2.8 prediction method was used.

### Generation of 3D Structures of Selected Epitopes

The PEP-FOLD3 online tool [21] was used to predict the 3D structures of the selected best epitopes [22-24].

### Molecular Docking and Molecular Dynamics Simulation

Molecular docking was carried out to depict the binding pattern of inhibitors with respective proteins. Predocking was carried out by UCSF Chimera [25]. The peptide-protein docking of the selected epitopes was carried out by the online docking tool PatchDock [26]. The results of PatchDock were refined and rescored by the FireDock server [27]. Docking was then performed by the HPEPDOCK server [28]. Docking pose analysis was performed using Ligplot [29]. The molecular simulation was executed with the GROMACS 2018.1 package using the Gromos43a1 force field [9]. Protein solvation was performed with the SPC water model in a cubic box (10.8 × 10.8 × 10.8 nm^3^). The solvated protein system was processed for energy minimization using the steepest algorithm up to a maximum of 25,000 steps or until the maximum force was not greater than 1000 kJ/mol/nm, which is the default threshold. The NVT and NPT ensembles for 50,000 steps (100 ps) were run at 300 K and 1 atm. The system was first equilibrated using the NVT ensemble followed by the NPT ensemble. The final molecular dynamic simulation was performed for the dock complex of the GVYFASTEK epitope docked against the HLA-A*11-01 allele (Protein Data Bank [PDB] ID 5WJL). Finally, the simulations were evaluated according to the root mean square deviation (RMSD) and root mean square fluctuation (RMSF) of atomic positions for the complete episode of simulations. All steps were similar across simulations, except that the final molecular dynamics simulation was carried out for 50 ns.

## Results

### Selection and Retrieval of Viral Protein Sequences

The SARS-CoV-2 strain was identified and 12 viral protein sequences against the human host in India were retrieved from the ViPR database and selected for possible vaccine candidate identification (Table 1). The FASTA sequences of the proteins are given in Multimedia Appendix 1.

**Table 1:** SARS-CoV-2 (Host: Human, Country: India) viral protein sequence identification and retrieval via the ViPR database.

Gene symbol	Protein name	GenBank nucleotide accession	GenBank protein accession
orf10	Orf10 protein	MT050493	QIA98591.1
orf8	Orf8 protein	MT050493	QIA98589.1
orf7a	Orf7a protein	MT050493	QIA98588.1
orf6	Orf6 protein	MT050493	QIA98587.1
orf3a	Orf3a protein	MT050493	QIA98584.1
M	Membrane glycoprotein	MT050493	QIA98586.1
E	Envelope protein	MT050493	QIA98585.1
S	Surface glycoprotein	MT050493	QIA98583.1
S	Surface glycoprotein	MT012098	QHS34546.1
N	Nucleocapsid protein	MT163715	QII87776.1
N	Nucleocapsid protein	MT163714	QII87775.1
N	Nucleocapsid phosphoprotein	MT050493	QIA98590.1

### Physicochemical Property Analysis and Protein Antigenicity

Analysis of physicochemical properties of the 12 proteins, including amino acids, molecular weight, theoretical isoelectric point (pI), extinction coefficient (M^-1^ cm^-1^), estimated half-life (in mammalian cells), instability index, aliphatic index, and grand average of hydropathicity (GRAVY), were predicted (Table 2). With a fixed threshold of 0.4, all proteins were predicted to be antigenic (Table 3). The physicochemical analysis revealed that the surface glycoprotein (QIA98583.1) had the highest extinction coefficient of 148,960 M^-1^ cm^-1^ and the lowest GRAVY value of –0.077 among the proteins. In addition, the surface glycoprotein was stable and antigenic; therefore, we selected this protein for further analysis.

**Table 2:** Physiochemical properties of SARS-CoV-2 viral proteins.

Gene symbol	Amino acids	Molecular weight	Theoretical pI^a^	Extinction coefficient (M^-1^ cm^-1^)	Half-life in mammalian cells (hours)	Instability index	Aliphatic index	GRAVY^b^
orf10	38	4449.23	7.93	4470	30	16.06 (stable)	107.63	0.637
orf8	121	13,804.93	5.42	16,305	30	46.24 (unstable)	94.13	0.181
orf7a	121	13,744.17	8.23	7825	30	48.66 (unstable)	100.74	0.318
orf6	61	7272.54	4.60	8480	30	31.16 (stable)	130.98	0.233
orf3a	275	31,122.94	5.55	58,705	30	32.96 (stable)	103.42	0.275
M	222	25,146.62	9.51	52,160	30	39.14 (stable)	120.86	0.446
E	75	8365.04	8.57	6085	30	38.68 (stable)	144.00	1.128
S	1273	141,206.52	6.24	148,960	30	33.01 (stable)	84.82	–0.077
S	1272	140,972.27	6.16	147,470	30	32.78 (stable)	85.05	–0.071
N	88	9827.08	10.23	8480	4.4	36.54 (stable)	61.14	–1.067
N	133	14,363.88	11.37	8480	1	58.97 (unstable)	44.21	–1.170
N	419	45,625.70	10.07	43,890	30	55.09 (unstable)	52.53	–0.971

^a^pI: isoelectric point.

^b^GRAVY: grand average of hydropathicity.

**Table 3:** Antigenicity prediction of SARS-CoV-2 viral proteins (threshold value: 0.4).

Protein name	Antigenicity score	Antigenicity
Orf10 protein	0.7185	Antigenic
Orf8 protein	0.6063	Antigenic
Orf7a protein	0.6441	Antigenic
Orf6 protein	0.6131	Antigenic
Orf3a protein	0.4945	Antigenic
Membrane glycoprotein	0.5102	Antigenic
Envelope protein	0.6025	Antigenic
Surface glycoprotein	0.4654	Antigenic
Surface glycoprotein	0.4687	Antigenic
Nucleocapsid protein	0.5767	Antigenic
Nucleocapsid protein	0.6235	Antigenic
Nucleocapsid phosphoprotein	0.5059	Antigenic

### T Cell and B Cell Epitope Prediction

**Figure 2:**
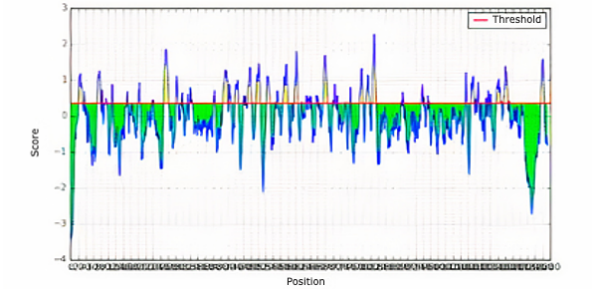
B cell epitope prediction for the surface glycoprotein of SARS-CoV-2 (QIA98583.1).

The T cell epitopes of MHC class I were determined by the NetMHCpan EL 4.0 prediction method of the IEDB server with the sequence length set to 9. The server-generated epitopes were further analyzed based on the antigenicity scores and percentile scores, and the top 10 potential epitopes were selected randomly for antigenicity, allergenicity, toxicity, and conservancy tests. The server ranks the predicted epitopes in ascending order of percentile scores (Table 4). The T cell epitopes of MHC class II (HLA-DRB1*04-01 allele) of the protein were also determined by the IEDB server (Table 5) using Sturniolo prediction methods. The top 10 ranked epitopes of the protein were selected randomly for further analysis. Additionally, the B cell epitopes of the protein were selected using the Bipipered linear epitope prediction method of the IEDB server, with the selection of epitopes based on greater lengths (Figure 2).

**Table 4:** Major histocompatibility complex class I epitopes of SARS-CoV-2 surface glycoprotein (QIA98583.1).

Epitope	Start	End	Topology	Antigenicity	Antigenicity score	Allergenicity	Toxicity	Minimum identity (%)	Conservancy (%)
GVYFASTEK	19	27	Inside	Yes	0.7112	Nonallergen	Nontoxic	11.11	100
VTYVPAQEK	15	23	Inside	Yes	0.8132	Allergen	Nontoxic	22.22	100
ASANLAATK	40	48	Inside	Yes	0.7041	Allergen	Nontoxic	22.22	100
TLADAGFIK	57	65	Inside	Yes	0.5781	Nonallergen	Nontoxic	22.22	100
TLKSFTVEK	22	30	Inside	No	0.0809	Allergen	Nontoxic	11.11	100
NSASFSTFK	20	28	Inside	No	0.1232	Allergen	Nontoxic	11.11	100
TEILPVSMTK	24	33	Inside	Yes	1.4160	Allergen	Nontoxic	10.00	100
SSTASALGK	29	37	Outside	Yes	0.6215	Allergen	Nontoxic	22.22	100
GTHWFVTQR	49	57	Inside	No	0.0723	Allergen	Nontoxic	11.11	100
EILPVSMTK	25	33	Inside	Yes	1.6842	Allergen	Nontoxic	11.11	100

**Table 5:** Major histocompatibility class II epitopes of SARS-CoV-2 surface glycoprotein (QIA98583.1).

Epitope	Start	End	Topology	Antigenicity	Antigenicity score	Allergenicity	Toxicity	Minimum identity (%)	Conservancy (%)
SNFRVQPTESI	36	46	Inside	Yes	0.9897	Allergen	Nontoxic	11.11	100
NFRVQPTESIV	37	47	Inside	Yes	1.0669	Nonallergen	Nontoxic	22.22	100
FRVQPTESIVR	38	48	Inside	No	0.3493	Allergen	Nontoxic	9.09	100
VYYHKNNKSWM	3	13	Inside	No	0.3726	Allergen	Nontoxic	18.18	100
LGVYYHKNNKS	1	11	Inside	Yes	0.8696	Allergen	Nontoxic	9.09	100
GVYYHKNNKSW	2	12	Inside	Yes	0.6685	Allergen	Nontoxic	9.09	100
LLIVNNATNVV	47	57	Inside	Yes	0.4166	Nonallergen	Nontoxic	9.09	100
LIVNNATNVVI	48	58	Inside	No	0.2045	Nonallergen	Nontoxic	9.09	100
IVNNATNVVIK	49	59	Inside	No	0.2274	Allergen	Nontoxic	9.09	100
VFVSNGTHWFV	44	54	Outside	No	0.0957	Allergen	Nontoxic	18.18	100

### Topology Identification of Epitopes

The topology of the selected epitopes was determined by the TMHMM v2.0 server. Table 4 and Table 5 represent the potential T-cell epitopes of selected surface glycoprotein. Table 6 shows the potential B cell epitopes with their respective topologies.

**Table 6:** B cell epitopes of SARS-CoV-2 surface glycoprotein (QIA98583.1).

Epitope	Topology	Antigenicity	Allergenicity
RTQLPPAYTNS	Inside	Antigen	Allergen
SGTNGTKRFDN	Inside	Antigen	Allergen
LTPGDSSSGWTAG	Outside	Antigen	Nonallergen
VRQIAPGQTGKIAD	Inside	Antigen	Nonallergen
YQAGSTPCNGV	Inside	Nonantigen	Nonallergen
QIAPGQTGKIAD	Inside	Antigen	Nonallergen
YGFQPTNGVGYQ	Outside	Antigen	Allergen
RDIADTTDAVRDPQ	Inside	Antigen	Allergen
QTQTNSPRRARSV	Inside	Nonantigen	Nonallergen
ILPDPSKPSKRS	Outside	Antigen	Nonallergen

### Antigenicity, Allergenicity, Toxicity, and Conservancy Analysis of Epitopes

The selected T cell epitopes were found to be highly antigenic as well as nonallergenic, nontoxic, and had a conservancy greater than 90%. Among the 10 selected MHC class I epitopes and 10 selected MHC class II epitopes, a total of four epitopes were selected based on the above-mentioned criteria: GVYFASTEK, TLADAGFIK, NFRVQPTESI, and LLIVNNATNV.

### Cluster Analysis of MHC Alleles

**Figure 3:**
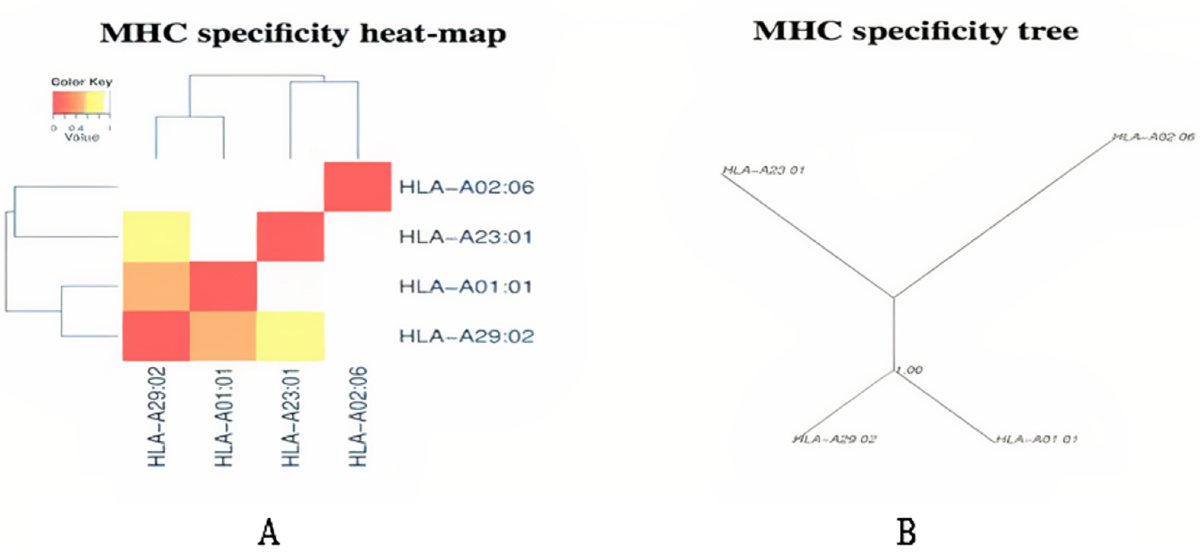
Major histocompatibility complex (MHC) class cluster analysis. (A) Heat map. (B) Specificity tree. The red zone indicates a strong interaction and the yellow zone corresponds to a weaker interaction.

The cluster analysis of the MHC class I alleles that possibly interact with the predicted epitopes was carried out by the online tool MHCcluster 2.0, which generates clusters of alleles phylogenetically. The results are shown in Figure 3, in which the red zone indicates a strong interaction and the yellow zone corresponds to a weaker interaction.

### Three-Dimensional Structure Prediction (Modeling) of Epitopes

**Figure 4:**
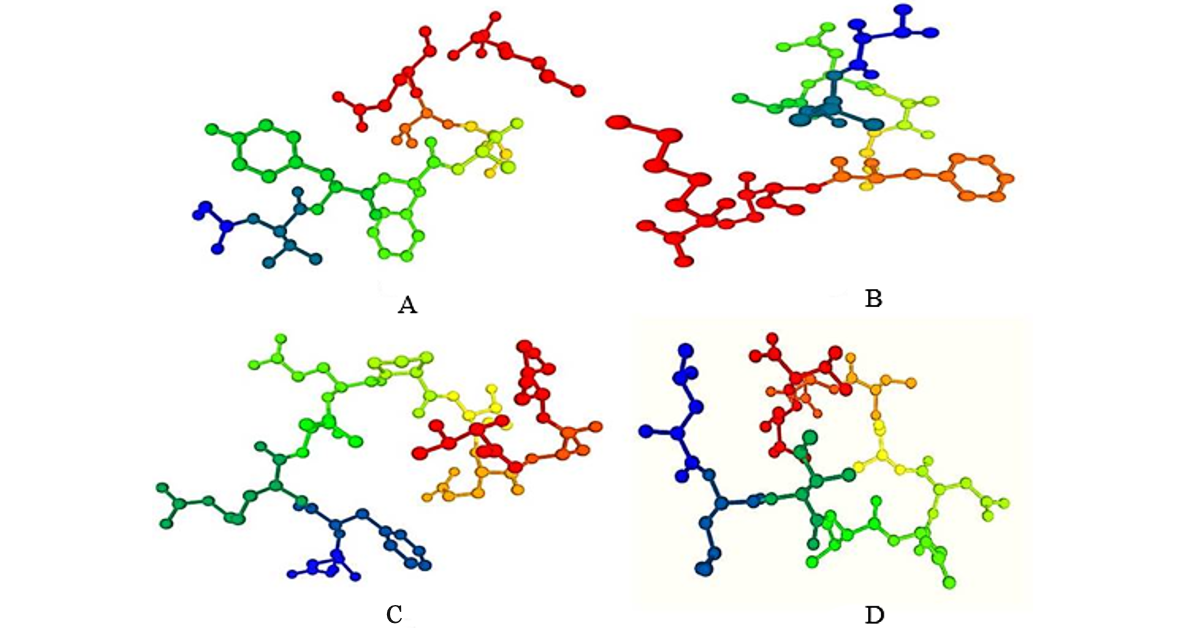
Three-dimensional structure generation of T-cell epitopes by the PEP-FOLD3 server. Epitope representation: (A) GVYFASTEK, (B) TLADAGFIK, (C) NFRVQPTESI, and (D) LLIVNNATNV.

All T cell epitopes were subjected to 3D structure prediction with the PEP-FOLD3 server, which were used for peptide-protein docking (Figure 4).

### Peptide-Protein Docking and Vaccine Candidate Prioritization

**Figure 5:**
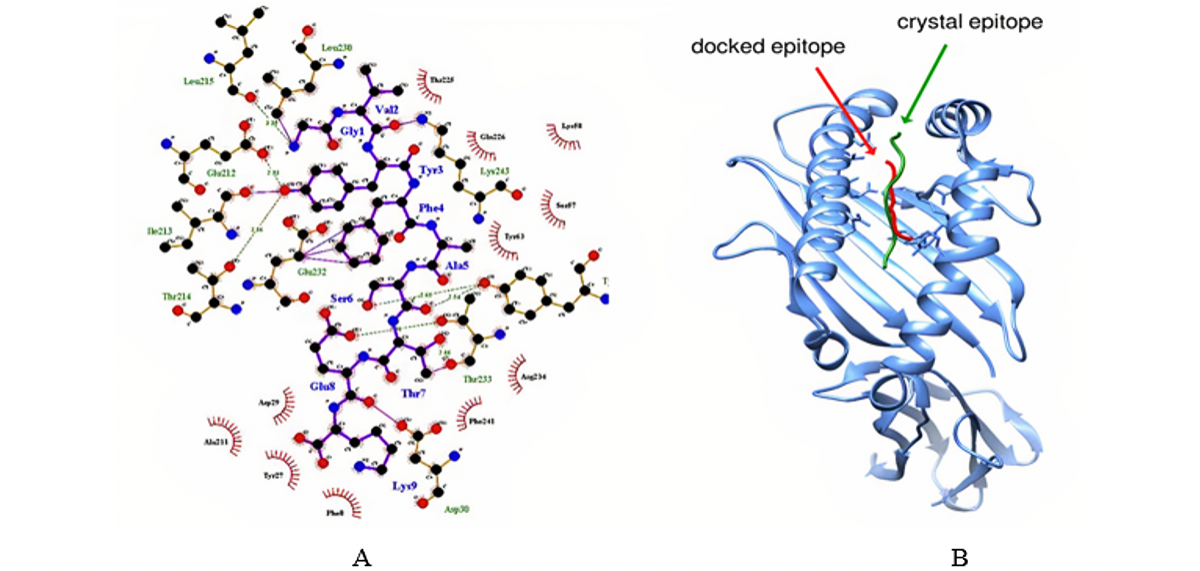
(A) Docking pose analysis via LigPlot (GVYFASTEK epitope docking against the HLA-A*11-01 allele [PDB ID: 5WJL]). Molecular docking result showing protein-ligand interaction. Oxygen (O), nitrogen (N), and carbon (C) atoms are represented by red, blue, and black circles, respectively. (B) Molecular docking analysis showing that the docking site of the ligand (GVYFASTEK epitope) in our study is similar to the ligand used in the crystal structure of the HLA-A*11-01 allele (PDB ID: 5WJL).

Molecular docking was performed to determine whether all of the identified epitopes could bind with MHC class I and MHC class II molecules. The selected epitopes docked with the HLA-A*11-01 allele (PDB ID 5WJL) and HLA-DRB1*04-01 allele (PDB ID 5JLZ). The docking was performed using the PatchDock online docking tool and refined by the FireDock online server. Results were also analyzed by the HPEPDOCK server (see Figure S1 in Multimedia Appendix 1). Among the four epitopes, the selected glycoprotein QIA98583.1, GVYFASTEK (MHC class I epitope), showed the best result with the lowest global energy of –52.82. Further, the docking pose was analyzed via Ligplot (Figure 5a) and the docking site can be visualized in Figure 5b. We also identified highly antigenic and nonallergenic B cell vaccine candidates LTPGDSSSGWTAG and VRQIAPGQTGKIAD from the selected surface glycoprotein (QIA98583.1).

### Molecular Dynamics Simulation

**Figure 6:**
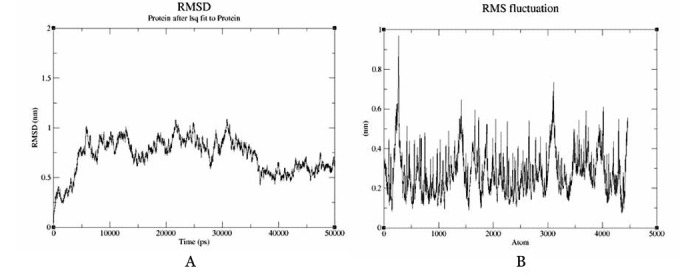
Molecular dynamics simulation. (A) Root mean square deviation (RMSD) and (B) root mean square fluctuation (RMSF) graphs of the dock complex (GVYFASTEK epitope docked against the HLA-A*11-01 allele [PDB ID: 5WJL]).

Molecular dynamics simulation of the dock complex of the GVYFASTEK epitope docked against the HLA-A*11-01 allele (PDB ID 5WJL) was successfully executed for 50 ns. The complex became stable throughout the simulation with an RMSD fluctuation of 0.3-1.0 nm from the original position (Figure 6a). In most cases, residues lying in the core protein regions have low RMSF values while exposed loops have high RMSF values (Figure 6b). The peaks in the graph show a value between 0.1 and 0.6 nm. Both these results indicate that the protein complexes were stable throughout the molecular docking simulations, demonstrating that the proteins possess good ability for stability.

## Discussion

### Principal Findings

A vaccine is an enormously imperative and expansively formed therapeutic product. Millions of infants, children, and adults are vaccinated every year. However, the development and research processes of vaccines are expensive and occasionally require countless months to prepare and advance an appropriate vaccine candidate toward eliminating a pathogen. There are currently innumerable tools and approaches of immunoinformatics, computer-aided drug design, bioinformatics, and converse/reverse vaccinology to extensively progress vaccine design and preparations, which in turn help to reduce the duration and cost investment for vaccine expansion [8,30].

In this study, physicochemical analysis revealed that the SARS-CoV-2 surface glycoprotein QIA98583.1 exhibited the highest extinction coefficient of 148,960 M^-1^ cm^-1^ and the lowest GRAVY value of –0.077 among the identified viral proteins. In addition, this selected surface glycoprotein was highly stable (instability index <40) and antigenic. The antigenicity of the protein was determined by the VaxiJen V2.0 server. If a compound has a variability index greater than 40, it means that the product is considered to be unbalanced [31]. The extinction coefficient refers to the quantity of light that is captured by a complex at a particular wavelength [32,33]. Various physicochemical properties, including the number of amino acids, molecular mass/weight, theoretical pI, extinction coefficient, uncertainty index, aliphatic index, and GRAVY, were resolved by the ProtParam server [34].

The two major functioning immune cells are B and T lymphocytic cells, which are responsible for several defensive roles in the body. Once identified by an antigen-presenting cell (APC; eg, dendritic cells and macrophages), the antigen is accessible by the MHC class II molecule existing on the surface of APCs to helper T cells. Subsequently, the helper T cell acquires a CD4+ fragment on its surface, designated as a CD4+ T cell. Once stimulated by an APC, helper T cells subsequently stimulate B cells, yielding antibody-producing plasma B cells alongside memory B cells. Plasma B cells harvest several antibodies and memory B cells function in long-term immunological memory. Moreover, macrophages and CD8+ cytotoxic T cells are also triggered by helper T cells to ultimately abolish the target antigen [35-39].

The possible B and T cell epitopes of the selected SARS-CoV-2 viral protein were identified by the IEDB server [14], which generates and ranks the epitopes based on their antigenicity scores and percentile scores. The top 10 MHC class I and class II epitopes were engaged for this investigation. The topology of the precise epitopes was resolved by the TMHMM v2.0 server [17]. In all inflammatory situations such as allergenicity, antigenicity, toxicity, and conservancy examinations, the T cell epitopes were found to be exceedingly antigenic with a higher immune response without allergenicity or toxicity, and showed a conservancy of over 90%. Among the 10 certain MHC class I and 10 selected MHC class II epitopes of the protein, four epitopes were designated based on the revealed properties, GVYFASTEK, TLADAGFIK, NFRVQPTESI, and LLIVNNATNVV, along with antigenic and nonallergenic B cell epitopes that were selected for additional vaccine candidate investigation. Cluster examination of the conceivable MHC class I and MHC class II alleles that might interact with the predicted epitopes was performed by the online tool MHC cluster 2.0 [20]. The antigenicity, demarcated as the capability of an extraneous ingredient to act as an antigen and stimulate B and T cell responses over their epitope, correspondingly identifies the antigenic determinant portion [40]. The allergenicity is defined as the capability of that ingredient to act as an allergen and induce latent allergic responses in the host [41].

Moreover, cluster analysis of the MHC class I and II alleles was similarly performed to categorize their association with each other and group them based on their functionality and predicted specificity [19]. In the following steps, peptide-protein docking was performed among the selected epitopes and MHC alleles. The MHC class I epitopes remained docked to the MHC class I molecule (PDB ID 5WJL) and the MHC class II epitopes were docked to the MHC class II molecule (PDB ID 5JLZ) correspondingly. The peptide-protein docking was performed to evaluate the capability of the epitopes to interact with the MHC molecules. Predocking was performed by UCSF Chimera and then 3D structure generation of the epitopes was performed. The docking was executed by the PatchDock and FireDock servers and analyzed by the HPEPDOCK server constructed on global energy. The GVYFASTEK epitope demonstrated the best scores in the peptide-protein docking. All of the vaccine candidates proved to be potentially antigenic and nonallergenic, indicating that they should not cause any allergenic reaction within the host. However, more in vitro and in vivo examinations should be performed to confirm the safety, usefulness, and potential of the predicted vaccine candidates.

### Conclusion

In the face of the enormous tragedy of suffering, demise, and social adversity caused by the COVID-19 pandemic, it is of extreme importance to develop an effective and safe vaccine against this disease. Bioinformatics, reverse vaccinology, and related technologies are widely used in vaccine design and development, since these technologies reduce costs and time. In this study, we first identified proteins belonging to SARS-CoV-2 against the human host from strains in India. The potential B cell and T cell epitopes that can effectively elicit cellular-mediated immune responses related to these selected proteins were then determined through robust processes. The potential T cell epitope (GVYFASTEK) and B cell epitopes (LTPGDSSSGWTAG, VRQIAPGQTGKIAD, QIAPGQTGKIAD, and ILPDPSKPSKRS) can play major roles in the development of new subunit and multiepitope vaccines. In brief, reverse vaccinology is confirmed as a reliable means to recognize novel vaccine candidates and their consequential application. This study can motivate further research in an innovative and efficient direction to deliver a fast, reliable, and significant platform in search of an effective and timely cure of COVID-19 caused by SARS-CoV-2.

## Appendix

Multimedia Appendix 1 SARS-CoV-2 protein sequences in FASTA format and HPEPDOCK server docking results (Figure S1).

## Abbreviations

APC: antigen-presenting cell
GRAVY: grand average of hydropathicity
HLA: human leukocyte antigen
IEDB: Immune Epitope Database
MHC: major histocompatibility complex
PDB: Protein Data Bank
pI: isoelectric point
RMSD: root mean square deviation
RMSF: root mean square fluctuation
WHO: World Health Organization
