# Supplementary material for: Identification of Potential Vaccine Candidates Against SARS-CoV-2 to Fight COVID-19: Reverse Vaccinology Approach
Source: JMIR Bioinform Biotechnol. 2022 Apr 26;3(1):e32401. doi: 10.2196/32401 (PMC9048139; doi:10.2196/32401)
Supplement: Multimedia Appendix 1 [file bioinform_v3i1e32401_app1.docx]

**Multimedia Appendix 1. SARS-CoV-2 protein sequences in FASTA format and HPEPDOCK server docking results (Figure S1).**

**FASTA FORMAT of SARS-CoV-2 proteins**

>QIA98591.1 orf10 protein [Severe acute respiratory syndrome coronavirus 2]

MGYINVFAFPFTIYSLLLCRMNSRNYIAQVDVVNFNLT

>QIA98589.1 orf8 protein [Severe acute respiratory syndrome coronavirus 2]

MKFLVFLGIITTVAAFHQECSLQSCTQHQPYVVDDPCPIHFYSKWYIRVGARKSAPLIELCVDEAGSKSP

IQYIDIGNYTVSCSPFTINCQEPKLGSLVVRCSFYEDFLEYHDVRVVLDFI

>QIA98588.1 orf7a protein [Severe acute respiratory syndrome coronavirus 2]

MKIILFLALITLATCELYHYQECVRGTTVLLKEPCSSGTYEGNSPFHPLADNKFALTCFSTQFAFACPDG

VKHVYQLRARSVSPKLFIRQEEVQELYSPIFLIVAAIVFITLCFTLKRKTE

>QIA98587.1 orf6 protein [Severe acute respiratory syndrome coronavirus 2]

MFHLVDFQVTIAEILLIIMRTFKVSIWNLDYIINLIIKNLSKSLTENKYSQLDEEQPMEID

>QIA98584.1 orf3a protein [Severe acute respiratory syndrome coronavirus 2]

MDLFMRIFTIGTVTLKQGEIKDATPSDFVRATATIPIQASLPFGWLIVGVALLAVFQSASKIITLKKRWQ

LALSKGVHFVCNLLLLFVTVYSHLLLVAAGLEAPFLYLYALVYFLQSINFVRIIMRLWLCWKCRSKNPLL

YDANYFLCWHTNCYDYCIPYNSVTSSIVITSGDGTTSPISEHDYQIGGYTEKWESGVKDCVVLHSYFTSD

YYQLYSTQLSTDTGVEHVTFFIYNKIVDEPEEHVQIHTIDGSSGVVNPVMEPIYDEPTTTTSVPL

>QIA98586.1 membrane glycoprotein [Severe acute respiratory syndrome coronavirus 2]

MADSNGTITVEELKKLLEQWNLVIGFLFLTWICLLQFAYANRNRFLYIIKLIFLWLLWPVTLACFVLAAV

YRINWITGGIAIAMACLVGLMWLSYFIASFRLFARTRSMWSFNPETNILLNVPLHGTILTRPLLESELVI

GAVILRGHLRIAGHHLGRCDIKDLPKEITVATSRTLSYYKLGASQRVAGDSGFAAYSRYRIGNYKLNTDH

SSSSDNIALLVQ

>QIA98585.1 envelope protein [Severe acute respiratory syndrome coronavirus 2]

MYSFVSEETGTLIVNSVLLFLAFVVFLLVTLAILTALRLCAYCCNIVNVSLVKPSFYVYSRVKNLNSSRV

PDLLV

>QIA98583.1 surface glycoprotein [Severe acute respiratory syndrome coronavirus 2]

MFVFLVLLPLVSSQCVNLTTRTQLPPAYTNSFTRGVYYPDKVFRSSVLHSTQDLFLPFFSNVTWFHAIHV

SGTNGTKRFDNPVLPFNDGVYFASTEKSNIIRGWIFGTTLDSKTQSLLIVNNATNVVIKVCEFQFCNDPF

LGVYYHKNNKSWMESEFRVYSSANNCTFEYVSQPFLMDLEGKQGNFKNLREFVFKNIDGYFKIYSKHTPI

NLVRDLPQGFSALEPLVDLPIGINITRFQTLLALHRSYLTPGDSSSGWTAGAAAYYVGYLQPRTFLLKYN

ENGTITDAVDCALDPLSETKCTLKSFTVEKGIYQTSNFRVQPTESIVRFPNITNLCPFGEVFNATRFASV

YAWNRKRISNCVADYSVLYNSASFSTFKCYGVSPTKLNDLCFTNVYADSFVIRGDEVRQIAPGQTGKIAD

YNYKLPDDFTGCVIAWNSNNLDSKVGGNYNYLYRLFRKSNLKPFERDISTEIYQAGSTPCNGVEGFNCYF

PLQSYGFQPTNGVGYQPYRVVVLSFELLHAPATVCGPKKSTNLVKNKCVNFNFNGLTGTGVLTESNKKFL

PFQQFGRDIADTTDAVRDPQTLEILDITPCSFGGVSVITPGTNTSNQVAVLYQDVNCTEVPVAIHADQLT

PTWRVYSTGSNVFQTRAGCLIGAEHVNNSYECDIPIGAGICASYQTQTNSPRRARSVASQSIIAYTMSLG

AENSVAYSNNSIAIPTNFTISVTTEILPVSMTKTSVDCTMYICGDSTECSNLLLQYGSFCTQLNRALTGI

AVEQDKNTQEVFAQVKQIYKTPPIKDFGGFNFSQILPDPSKPSKRSFIEDLLFNKVTLADAGFIKQYGDC

LGDIAARDLICAQKFNGLTVLPPLLTDEMIAQYTSALLAGTITSGWTFGAGAALQIPFAMQMAYRFNGIG

VTQNVLYENQKLIANQFNSVIGKIQDSLSSTASALGKLQDVVNQNAQALNTLVKQLSSNFGAISSVLNDI

LSRLDKVEAEVQIDRLITGRLQSLQTYVTQQLIRAAEIRASANLAATKMSECVLGQSKRVDFCGKGYHLM

SFPQSAPHGVVFLHVTYVPAQEKNFTTAPAICHDGKAHFPREGVFVSNGTHWFVTQRNFYEPQIITTDNT

FVSGNCDVVIGIVNNTVYDPLQPELDSFKEELDKYFKNHTSPDVDLGDISGINASVVNIQKEIDRLNEVA

KNLNESLIDLQELGKYEQYIKWPWYIWLGFIAGLIAIVMVTIMLCCMTSCCSCLKGCCSCGSCCKFDEDD

SEPVLKGVKLHYT

>QHS34546.1 surface glycoprotein [Severe acute respiratory syndrome coronavirus 2]

MFVFLVLLPLVSSQCVNLTTRTQLPPAYTNSFTRGVYYPDKVFRSSVLHSTQDLFLPFFSNVTWFHAIHV

SGTNGTKRFDNPVLPFNDGVYFASTEKSNIIRGWIFGTTLDSKTQSLLIVNNATNVVIKVCEFQFCNDPF

LGVYHKNNKSWMESEFRVYSSANNCTFEYVSQPFLMDLEGKQGNFKNLREFVFKNIDGYFKIYSKHTPIN

LVRDLPQGFSALEPLVDLPIGINITRFQTLLALHRSYLTPGDSSSGWTAGAAAYYVGYLQPRTFLLKYNE

NGTITDAVDCALDPLSETKCTLKSFTVEKGIYQTSNFRVQPTESIVRFPNITNLCPFGEVFNATRFASVY

AWNRKRISNCVADYSVLYNSASFSTFKCYGVSPTKLNDLCFTNVYADSFVIRGDEVIQIAPGQTGKIADY

NYKLPDDFTGCVIAWNSNNLDSKVGGNYNYLYRLFRKSNLKPFERDISTEIYQAGSTPCNGVEGFNCYFP

LQSYGFQPTNGVGYQPYRVVVLSFELLHAPATVCGPKKSTNLVKNKCVNFNFNGLTGTGVLTESNKKFLP

FQQFGRDIADTTDAVRDPQTLEILDITPCSFGGVSVITPGTNTSNQVAVLYQDVNCTEVPVAIHADQLTP

TWRVYSTGSNVFQTRAGCLIGAEHVNNSYECDIPIGAGICASYQTQTNSPRRARSVASQSIIAYTMSLGA

ENSVAYSNNSIAIPTNFTISVTTEILPVSMTKTSVDCTMYICGDSTECSNLLLQYGSFCTQLNRALTGIA

VEQDKNTQEVFAQVKQIYKTPPIKDFGGFNFSQILPDPSKPSKRSFIEDLLFNKVTLADAGFIKQYGDCL

GDIAARDLICAQKFNGLTVLPPLLTDEMIAQYTSALLAGTITSGWTFGAGAALQIPFAMQMAYRFNGIGV

TQNVLYENQKLIANQFNSAIGKIQDSLSSTASALGKLQDVVNQNAQALNTLVKQLSSNFGAISSVLNDIL

SRLDKVEAEVQIDRLITGRLQSLQTYVTQQLIRAAEIRASANLAATKMSECVLGQSKRVDFCGKGYHLMS

FPQSAPHGVVFLHVTYVPAQEKNFTTAPAICHDGKAHFPREGVFVSNGTHWFVTQRNFYEPQIITTDNTF

VSGNCDVVIGIVNNTVYDPLQPELDSFKEELDKYFKNHTSPDVDLGDISGINASVVNIQKEIDRLNEVAK

NLNESLIDLQELGKYEQYIKWPWYIWLGFIAGLIAIVMVTIMLCCMTSCCSCLKGCCSCGSCCKFDEDDS

EPVLKGVKLHYT

>QII87776.1 nucleocapsid protein, partial [Severe acute respiratory syndrome coronavirus 2]

AALALLLLDRLNQLESKMSGKGQQQQGQTVTKKSAAEASKKPRQKRTATKAYNVTQAFGRRGPEQTQGNFGDQELIRQGTDYKHWPQI

>QII87775.1 nucleocapsid protein, partial [Severe acute respiratory syndrome coronavirus 2]

RGGSQASSRSSSRSRNSSRNSTPGSSKRTSPARMAGNGGDAALALLLLDRLNQLESKMSGKGQQQQGQTV

TKKSAAEASKKPRQKRTATKAYNVTQAFGRRGPEQTQGNFGDQELIRQGTDYKHWPQIAQFAP

>QIA98590.1 nucleocapsid phosphoprotein [Severe acute respiratory syndrome coronavirus 2]

MSDNGPQNQRNAPRITFGGPSDSTGSNQNGERSGARSKQRRPQGLPNNTASWFTALTQHGKEDLKFPRGQ

GVPINTNSSPDDQIGYYRRATRRIRGGDGKMKDLSPRWYFYYLGTGPEAGLPYGANKDGIIWVATEGALN

TPKDHIGTRNPANNAAIVLQLPQGTTLPKGFYAEGSRGGSQASSRSSSRSRNSSRNSTPGSSRGTSPARM

AGNGGDAALALLLLDRLNQLESKMSGKGQQQQGQTVTKKSAAEASKKPRQKRTATKAYNVTQAFGRRGPEQTQGNFGDQELIRQGTDYKHWPQIAQFAPSASAFFGMSRIGMEVTPSGTWLTYTGAIKLDDKDPNFKDQVILLNKHIDAYKTFPPTEPKKDKKKKADETQALPQRQKKQQTVTLLPAADLDDFSKQLQQSMSSADSTQA


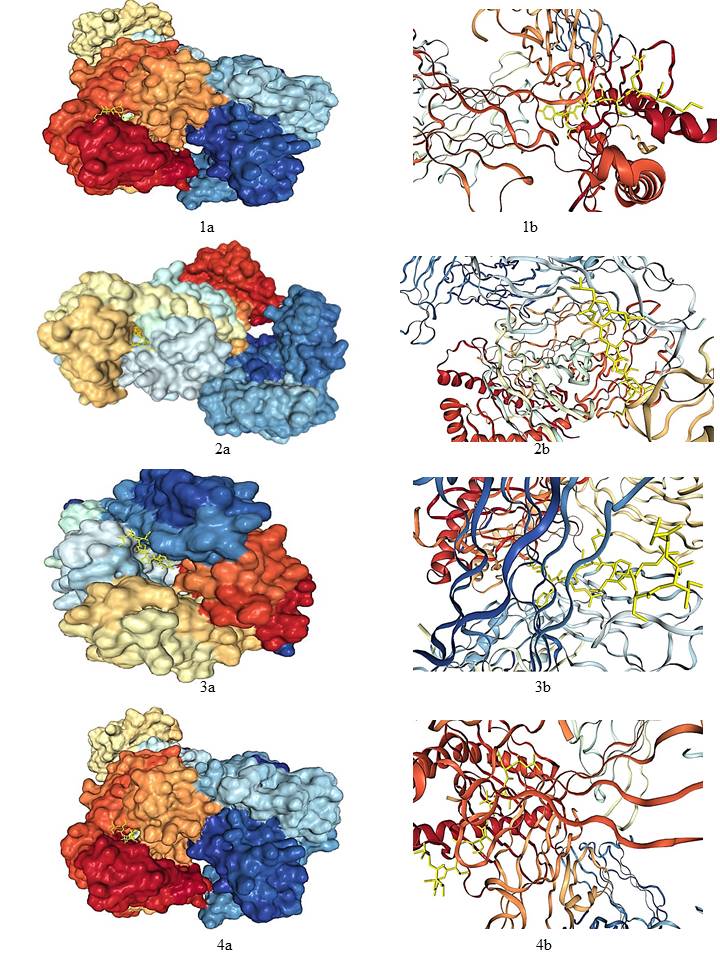


***Fig S1. HPEPDOCK server docking results, where (a: surface view of peptide-protein docking, b: Licorice view of peptide-protein docking) (1: GVYFASTEK, 2: TLADAGFIK, 3: NFRVQPTESI,4: LLIVNNATNV)***
